# Bacterial Cross-Contamination in a Veterinary Ophthalmology Setting

**DOI:** 10.3389/fvets.2020.571503

**Published:** 2020-12-23

**Authors:** Dominic Gentile, Rachel A. Allbaugh, Mehmet C. Adiguzel, Danielle E. Kenne, Orhan Sahin, Lionel Sebbag

**Affiliations:** ^1^Department of Veterinary Clinical Sciences, College of Veterinary Medicine, Iowa State University, Ames, IA, United States; ^2^Department of Veterinary Microbiology and Preventive Medicine, College of Veterinary Medicine, Iowa State University, Ames, IA, United States; ^3^Department of Microbiology, Faculty of Veterinary Medicine, Ataturk University, Erzurum, Turkey; ^4^Department of Veterinary Diagnostic and Production Animal Medicine, College of Veterinary Medicine, Iowa State University, Ames, IA, United States

**Keywords:** nosocomial infection, healthcare-associated infection, environmental contamination, ophthalmic equipment, pulse-field gel electrophoresis, bacterial keratitis

## Abstract

The present study describes the prevalence of bacterial cross-contamination in a veterinary ophthalmology setting, a serious issue that can result in healthcare-associated (or nosocomial) infections among patients and staff. Retrospective (*n* = 5 patients) and prospective (*n* = 23 patients) studies evaluated bacterial isolates in companion animals presenting with ulcerative keratitis, sampling the patients' cornea and surrounding examination room, including the environment (exam table, countertop, floor) and ophthalmic equipment (slit lamp, transilluminator, direct ophthalmoscope, indirect headset, tonometer). Results of bacterial culture and antibiotic susceptibility testing were recorded, and degree of genetic relatedness was evaluated in six pairs of isolates (cornea + environment or equipment) using pulse-field gel electrophoresis (PFGE). Overall contamination rate of ophthalmic equipment, environment, and examination rooms (equipment + environment) was 42.9% (15/35 samples), 23.7% (9/38 samples) and 32.9% (24/73 samples), respectively. Methicillin-resistant *Staphylococcus pseudintermedius* (MRSP), a multi-drug resistant (MDR) pathogen with zoonotic potential, was isolated in 8.2% (6/73) of samples. The patient's cornea was likely the source of cross-contamination in 50% (3/6) of MRSP pairs as evaluated by PFGE; notably, two of the three similar bacterial strains did not have an exact match of their antibiotic susceptibility profiles, highlighting the importance of advanced diagnostics such as PFGE to assess cross-contamination in healthcare facilities. Future work could examine the contamination prevalence of specific equipment or the efficacy of cleaning protocols to mitigate cross-contamination in veterinary practice.

## Introduction

Cross-contamination, or the transfer of pathogens from patients to the environment and vice versa, is a serious concern in veterinary and human medicine. Infectious pathogens can survive in the environment for extended periods. A systematic review by Kramer et al. showed that most gram-positive bacteria can survive for months on a dry inanimate surface, while gram-negative bacteria can subsist in the environment for longer durations (1). Environmental pathogens can contaminate individuals and result in healthcare-associated (or nosocomial) infections, with the potential to cause detrimental effects to the patient. For instance, contaminated eyedrops resulted in 13 cases of post-operative endophthalmitis after uneventful cataract surgery in one report (2), and development of bacterial keratitis in three patients in another report (3). On both occasions, indistinguishable pathogens were cultured from the patient's eyes and the contaminated environmental source.

In ophthalmology, sources of contamination include the surrounding environment (e.g., countertops) but also examination and diagnostic equipment commonly used by healthcare staff. In the human medicine literature, the contamination prevalence of ophthalmic materials ranges from 11.7% for ophthalmic solutions (4), 28% for ophthalmic medications (5), 54% for slit lamps (6), 76% for handheld lenses (6), and 82% for sterile ultrasound biomicroscopy probes (7). Similar information for diagnostic equipment in the veterinary field is limited to a single report describing a contamination prevalence of 58.6% (17/29) for slit lamps used in a veterinary ophthalmic practice (8).

The primary goal of the current study was to describe the prevalence of environmental contamination in a veterinary ophthalmology practice using a retrospective and prospective study design. Given the frequent occurrence of bacterial keratitis in veterinary patients, a secondary objective was to evaluate the molecular relatedness between bacteria isolated from the cornea of patients and the ones isolated from their surrounding environment. Together, we hope this study will heighten awareness and provide guidance to mitigate cross-contamination in veterinary ophthalmology, most notably after clinical examination of companion animals with a potentially infected ocular surface.

## Materials and Methods

### Retrospective Investigation

Since January 2015, a standard operating protocol (SOP) was initiated at our institution for every clinical patient from which a multi-drug resistant (MDR) bacteria was cultured. For dogs and cats evaluated by the ophthalmology service, the SOP consisted of using a moistened sterile culturette swab for each of the following sites where the patient was examined: (i) Environment swabs, including the front aspect of the countertop (facing the docking station of the slit lamp), the front aspect of the examination table, the floor area in front of the examination table, and the inside and door of the cage housing the patient if the patient was hospitalized overnight; and (ii) Equipment swabs, including the handheld slit lamp (SL-15 or SL-17, Kowa Company Ltd.; magnification and illumination dials), rebound tonometer (TonoVet, ICare Finland; measuring and selector buttons), and indirect headset (Keeler Vantage Plus). After environmental sampling and before another patient was evaluated, the examination room was disinfected following the hospital's protocol. Protective gloves and outerwear were donned to protect all exposed skin, and gross debris was removed from surfaces to be disposed of in a biohazard bag. An accelerated hydrogen peroxide-based disinfectant (Accel/Rescue^TM^, Virox Animal Health) was applied to all areas previously described (equipment, countertop, examination table, floor) for the labeled contact time before being wiped clean with paper towels. Medical records were reviewed from January 2015 to February 2019, recording the bacteria isolated only from clinical patients with confirmed MDR infections as well as the results of subsequent cultures from the environment and ophthalmic equipment.

### Prospective Investigation

The prospective experiment extended from March 2019 to April 2020 and involved the collection of environmental and equipment cultures at the end of the working day, evaluating examination rooms in which a companion animal was diagnosed with any suspected infectious keratitis. A corneal swab from the patient was also submitted for aerobic culture and antimicrobial susceptibility testing. Due to logistical constraints, the prospective study was limited to 23 clinical patients – haphazardly selected based on personnel availability and patients presenting during working hours – and two culturette swabs from the exam room for each case. The first swab was labeled “environment” and included the front aspect of the countertop (facing the docking station of the slit lamp), the front aspect of the examination table, and the floor area in front of the examination table. The second swab was labeled “equipment” and included the handheld slit lamp (SL-15 or SL-17, Kowa Company Ltd.; magnification and illumination dials), rebound tonometer (TonoVet, ICare Finland; measuring and selector buttons), the light switch of the Finoff transilluminator (Welch Allyn), direct ophthalmoscope (Welch Allyn), and indirect headset (Keeler Vantage Plus). After each patient, the floor was swept to remove gross debris and the exam table was wiped clean with Accel/Rescue^TM^ and paper towels. Ophthalmology staff was aware of the nature of the study, although no changes in routine examination techniques or cleaning protocols were implemented.

### Microbiologic Sample Isolation, Identification, and Susceptibility Testing

Sterile culturette swabs (BBL^TM^ CultureSwab^TM^, Becton Dickinson and Company, Sparks, MD) were used to obtain samples for all bacterial cultures. In clinical patients, corneal cultures were collected by anesthetizing the ocular surface (0.5% propracaine ophthalmic solution) followed by gentle rub of the pre-moistened swab tip against the edges of the corneal defect, with care not to touch the conjunctiva or eyelids to avoid contamination. As previously described (9), corneal samples were processed for aerobic microbiologic assessment *via* inoculation of a non-selective medium [tryptic soy agar with 5% sheep blood (Blood Agar, Hardy Diagnostics, catalog #A10)] and a Gram-negative selective medium (MacConkey Agar, Hardy Diagnostics, catalog #G35). The blood agar was incubated at 35°C ± 2°C with 5–10% CO_2_ for a total length of 4 days while the MacConkey agar was incubated 35°C ± 2°C without CO_2_ for a total length of 2 days. Both agar plates were observed for growth every 24 h. Organisms were then identified using Matrix-Assisted Laser Desorption Ionization Time-of-Flight mass-spectrometry (MALDI-TOF MS, Bruker) or conventional microbiology methods if necessary. Minimum inhibitory concentration (MIC) susceptibility testing was performed using an automated broth microdilution system (Sensititre AIM, Trek Diagnostic System Inc.) and susceptibility panels (Thermo Fisher Scientific). Interpretations were determined by the MIC breakpoints, which are based on the VET08 and M100 Clinical and Laboratory Standards Institute (CLSI) documents (10, 11). Susceptibility testing included an ophthalmic susceptibility profile (JOEYE2 plate, Thermo Scientific Inc.) and/or a systemic susceptibility profile. Bacterial isolates were considered multidrug resistant (MDR) if resistant to three or more classes of antibiotics (12), removing all known intrinsic resistances from the MDR definition as described by Sweeney et al. (13).

### Pulse-Field Gel Electrophoresis

Based on the results of bacterial cultures and susceptibility profiles, six pairs (five patients) of corneal isolates and associated environmental or equipment isolates were assessed for genetic relatedness testing. Analysis of macrorestriction fragment patterns of *Staphylococcus pseudintermedius* genomic DNA using SmaI restriction enzyme was performed following previously described methods (14–16) with minor modifications. *Staphylococcus* isolates retrieved from −80°C freezer were incubated overnight at 35°C on Blood Agar containing 5% sheep blood (Remel, Lenexa, KS). A well-isolated single colony from each single-isolate agar plate was transferred to Mueller Hinton broth (Becton-Dickinson, Sparks, MD, USA) and incubated overnight at 35°C. Those cultures were embedded in 1.2% Seakem Gold agarose (Lonza, Rockland, ME, USA) and treated with lysostaphin at a concentration of 1 mg/mL (Sigma-Aldrich, St Louis, Missouri, USA) for 1 h at 37°C in a shaking water bath, followed by incubation for 30 min at 50°C in a static water bath in proteinase K at a concentration of 0.1 mg/ml (Sigma-Aldrich). Gel plugs were washed and digested with SmaI overnight at 25°C then all plugs were embedded in the same 1.2% Seakem Gold agarose gel (Lonza). DNA fragments were separated using the CHEF Mapper electrophoresis system (Bio-Rad, Hercules, CA) in 0.5×TBE buffer (at conditions 14°C, 6 V/cm, initial switching time from 5–15 s for 8.5 h and final switching time from 15–60 s for 11.5 h), the gel was stained with ethidium bromide for 30 min, and photographed by ChemiDoc Gel Imaging System (Bio-Rad, Hercules, CA, USA). PFGE patterns were analyzed by the GelCompare II v.6.5 software program (Applied Maths, Kortrijik, Belgium) using the Dice similarity coefficient and unweighted-pair group method with arithmetic averages (UPGMA) with 1% optimization and 1% position tolerance. Lambda DNA ladder (Bio-Rad) was used as the molecular size marker.

## Results

Table 1 summarizes all bacterial isolates from patients' corneas and the surrounding environment in examination rooms. Positive growth refers to a positive bacterial culture of a pathogenic bacteria. Bacteria likely considered contaminants (e.g., Bacillus, Micrococcus) were excluded from data analysis (i.e., percent of environmental contaminants), but were listed in Table 1 for completeness. The retrospective study involved five dogs with bacterial keratitis (5/5 or 100% positive growth), twelve equipment samples (5/12 or 41.7% positive growth), and fifteen environmental samples (6/15 or 40% positive growth), yielding an overall examination room contamination rate of 40.7% (11/27 samples). The prospective study involved 21 dogs and 2 cats with bacterial keratitis (11/23 or 47.8% positive growth), 23 equipment samples (10/23 or 43.5% positive growth), and 23 environmental samples (3/23 or 13% positive growth), yielding an overall examination room contamination rate of 28.3% (13/46 samples). Taken together, the contamination rates of ophthalmic equipment, environment, and examination rooms (equipment + environment) were 42.9% (15/35), 23.7% (9/38), and 32.9% (24/73), respectively.

**Table 1:** Summary of bacteria isolates from the cornea, environment, or equipment in the retrospective study (patients 1–5) and prospective study (patients 6–28).

**ID**	**Signalment**	**Cornea**	**Environment**	**Equipment**
1	7 m/o MI Saint Bernard	S. pseudintermedius (MDR, MRSP); *S. canis*	*S. pseudintermedius* (MDR, MRSP) (Floor)	*Staphylococcus aureus* (Slit lamp); *S. pseudintermedius* (TonoVet); Coagulase negative *Staphylococcus* group (MDR, MRSP) (Slit lamp)
2	12.5 y/o MC Cairn Terrier	*S. pseudintermedius* (MDR, MRSP)	*S. pseudintermedius* (MDR, MRSP) (Exam table)	Coagulase negative *Staphylococcus* group (MDR, MRSP) (TonoVet)
3	4 y/o MC French Bulldog	*S. pseudintermedius* (MDR, MRSP)	*S. pseudintermedius* (MDR, MRSP) (Floor)	*S. pseudintermedius* (MDR, MRSP) (Headset)
4	3 y/o FS mixed breed dog	*S. pseudintermedius* (MDR, MRSP); *Streptococcus* alpha haemolytic; Gram-negative rod	*S. pseudintermedius* (Cage); *S. pseudintermedius* (Cage)	–
5	6.5 y/o MC Pug	*S. pseudintermedius* (MDR, MRSP)	*Staphylococcus schleiferi* ss coagulans (Floor)	–
6	8 y/o FS mixed breed dog	S. pseudintermedius; *Streptococcus agalactiae*	*Streptococcus agalactiae*	S. pseudintermedius; *Bacillus* species
7	10 y/o MC mixed breed dog	S. pseudintermedius; *Corynebacterium* species; *S. pseudintermedius* (separate isolate)	S. pseudintermedius; *Staphylococcus aureus*	*Staphylococcus epidermidis*; *Staphylococcus haemolyticus*; *Bacillus* species; *Paenibacillus* species
8	14 y/o MC Schnauzer mixed	*S. canis*	–	*Psychrobacter* species; *Bacillus* species; *Aerococcus viridans*
9	12 y/o MC Shih Tzu	*P. aeruginosa*	–	*Streptomyces* species; *Micrococcus* species
10	11 y/o MC Dachshund	–	–	*Bacillus pumilus*
11	7 y/o FS Shih Tzu	Corynebacterium mastitidis	–	*Bacillus* species
12	13 y/o FS Shih Tzu	–	–	*Staphylococcus epidermidis*
13	7 y/o FS Boston Terrier	–	–	*Macrococcus* species; *Bacillus megaterium*; Coagulase negative *Staphylococcus* group
14	8 y/o FS French Bulldog	–	–	*Bacillus megaterium*
15	12 y/o MC mixed breed dog	–	–	–
16	11 y/o MI Boston Terrier	–	–	Coagulase negative *Staphylococcus* group; *Enterococcus faecium*; *S. pseudintermedius*
17	10 y/o FS mixed breed dog	*S. canis*; *S. pseudintermedius*; *Achromobacter xylosoxidans*	–	–
18	13 y/o FS Domestic Shorthair	*S. pseudintermedius* (MDR, MRSP)	–	*Bacillus cereus*; *S. canis*
19	6 y/o MC Shih Tzu	*P. aeruginosa*	–	–
20	9 y/o MC Boston Terrier	*S. canis*; Gram-negative non-fermenter	–	–
21	10 m/o FS English Bulldog	–	*S. pseudintermedius*; *Rothia* species; *Bacillus* species; *Bacillus thuringiensis*	–
22	8 y/o MC Persian cat	–	–	–
23	8 y/o MC Chihuahua	–	–	–
24	7 y/o FS Cavalier King Charles Spaniel	*P. aeruginosa*	–	–
25	13 y/o MC Shih Tzu	–	–	–
26	13 y/o MC Bichon Frise	–	–	–
27	2 y/o MI Pembroke Welsh Corgi	–	–	*Psychrobacter* species; *Corynebacterium auriscanis*
28	11 y/o FS Pug	*P. aeruginosa*	–	*Staphylococcus capitis*; *Staphylococcus epidermidis*; Gram negative oxidase negative rod; *Corynebacterium tuberculostearicum*; Gram positive catalase positive rod

**Figure 1:**
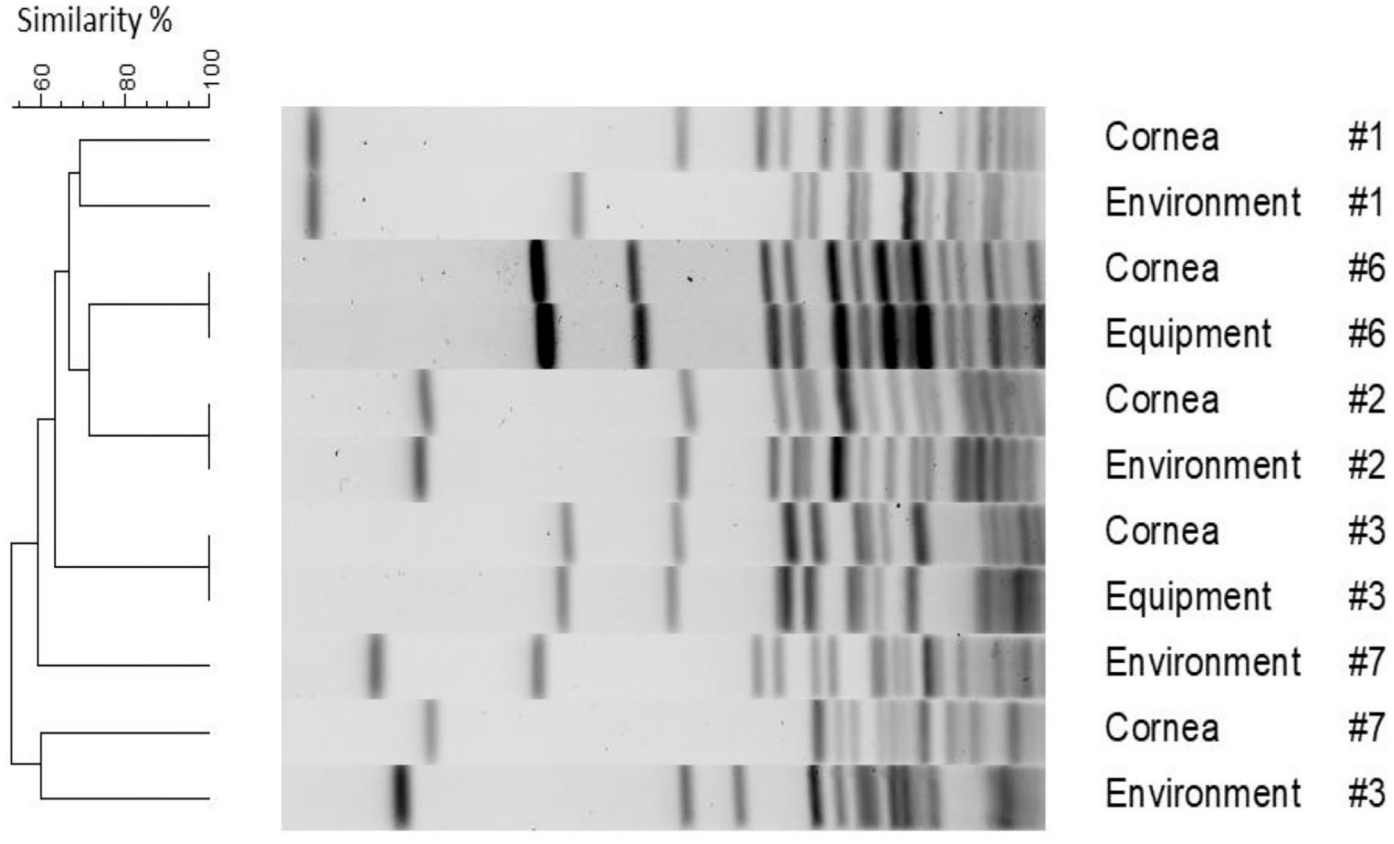
Pulse-field gel electrophoresis dendrogram highlighting the degree of genetic relatedness of bacteria isolated from five clinical patients and their surrounding environment. The dendrogram is constructed from the restriction patterns of SmaI-digested *S. pseudintermedius* genomes, with bacterial source and patient ID depicted next to the restriction profiles.

*Bacterial isolates highlighted in gray were analyzed further for genomic relatedness; the patients ID (first column) and sampling locations correspond to the descriptors next to the restriction profiles in Figure 1. – Indicates no growth on culture; MRSP, methicillin-resistant isolate; MDR, multi-drug resistant isolate*.

Examination room bacteria considered MDR represented 6/73 (8.2%) of all isolates, with 6/73 (8.2%) classified as methicillin-resistant *S. pseudintermedius* (MRSP).

Figure 1 depicts PFGE results for the six pairs of bacteria (five patients) that were assessed for genetic relatedness (patients #1, #2, #3, #6, and #7). PFGE was also completed for bacterial isolates associated with patient #4; however, results are not depicted in Figure 1 as the environmental isolate did not yield readable results despite multiple attempts, possibly due to resisting digestion with the restriction enzyme. Three out of six pairs (50%) were indistinguishable including corneal/environmental isolates for patient #2 and corneal/equipment isolates for patients #3 and #6; of note, the latter two pairs had slightly different antibiotic susceptibility profiles despite being confirmed indistinguishable on PFGE. The other three pairs (corneal/environment isolates for patients #1, #3, and #7) were highly genetically diverse, with ≤70% similarity level.

## Discussion

The present study evaluated the contamination rate of examination rooms in a veterinary ophthalmology setting, identifying the patients' corneal infection as the likely source of contamination in selected cases. The overall prevalence of contamination in ophthalmic exam rooms (environment and equipment) was 32.9% (24/73 samples) when results of the retrospective and prospective studies were combined. In comparison, environmental surveillance of various sites in 101 veterinary hospitals (e.g., reception, treatment rooms, kennels) detected a contamination prevalence that ranged from 2 to 92% depending on the bacterial species (17). Although the same information on ophthalmology exam rooms is not available in the human medicine literature, the authors believe the contamination rate is likely higher in veterinary ophthalmology exam rooms for two reasons: (i) increased risk for cross-contamination due to normal animal behaviors such as sniffing, self-grooming and laying on floors (17, 18); and (ii) increased hand contact between clinicians and patients in veterinary vs. human ophthalmology, with the need to manually open the eyelids and use handheld instruments (e.g., slit lamp, tonometer) in companion animals more frequently than in humans.

Many bacteria recovered in this study were potential pathogens that could place human and animal health at risk. For instance, *S. pseudintermedius* is a common pathogen in companion animals that also represents an emerging zoonotic pathogen in humans (19). Notably, methicillin-resistant *S. pseudintermedius* (MRSP) was of concern in the present report. Such isolates accounted for 8.2% (6/73) of all environmental samples, slightly higher than prior reports in veterinary medicine (6.4–7.0%) (17, 20), and MRSP represents pathogens that are particularly difficult to manage given their virulence factors and multi-drug resistance (21, 22).

Contamination of ophthalmic equipment ranged from 41.7% (5/12) to 43.5% (10/23) in the retrospective and prospective studies, respectively, with an overall prevalence of 42.9% (15/35). These findings are fairly similar to a recent report by Casola and colleagues, who detected pathogenic bacteria in 58.6% of handheld slit lamps used in a veterinary practice (8). It would be interesting to determine whether contamination is more common in select ophthalmic equipment. The study by Casola et al. solely evaluated slit lamps, while the current study only differentiated between separate equipment items for the retrospective study (12 samples), identifying positive growth in slit lamps (2/12), rebound tonometers (2/12), and indirect headset (1/12) with negative growth in the other samples (7/12).

Contamination of the environment ranged from 13.0% (3/23) to 40.0% (6/15) in the prospective and retrospective studies, respectively, with an overall prevalence of 23.7% (9/38). The lower contamination rate of environment vs. equipment was surprising, given the assumption that hard surfaces (floor, table, countertop) would be suitable substrates for bacterial colonization (23). Ophthalmic equipment may be particularly prone to contamination in veterinary medicine, given the aforementioned factors (e.g., eyelid manipulation, handheld instruments). Another explanation is the efficacy (or lack thereof) of cleaning protocols in place at the time of the study. In our practice, exam tables and countertops were cleaned with accelerated hydrogen peroxide after each consultation; however, individual equipment items were generally disinfected at the end of the working day.

Undoubtedly, proper cleaning protocols can reduce bacterial levels in examination rooms and minimize the risk for nosocomial infections (24, 25), as exemplified by a significant decrease in bacterial contamination of handheld slit lamps from 58.6 to 13.8% following disinfection (8).

The exact source of contamination can be challenging to determine. In the present study, genomic relatedness was assessed with PFGE for six pairs of associated bacteria isolated from veterinary patients' corneas and their surrounding environment. We showed that 3/6 (50%) pairs yielded indistinguishable molecular profiles on PFGE, potentially indicating direct contamination of the environment from the corneal infection in these three cases. The bacteria isolated in the exam room for the other cases did not match the organism cultured from the patient's cornea and may have originated from other anatomical sites from the same patient (e.g., skin, mouth, etc.), other patients examined in the same examination room, or healthcare staff involved in the animals' care. In fact, cross-contamination with identical pathogens not only occurs between animals and surrounding inanimate objects but also between animals and other living beings in the environment putting humans at risk for transmission of zoonotic pathogens (26–28). Importantly, the present work shows that antibiotic susceptibility profiles are not sufficient to characterize relatedness (or lack thereof) of two similar strains of bacteria isolated in the same examination room. Here, there were two cases (patient #3 and patient #6) in which PFGE identified molecularly indistinguishable bacteria, while the antibiotic susceptibility profiles of the similar strains differed. The discrepancies between antibiotic susceptibility profiles may arise from the integrity of the antibiotic disks used during the test or the subjectivity between technicians when precisely measuring zones of inhibition.

Several limitations inherent to the study design resulted in a reported contamination prevalence that is likely a gross underestimation of the actual rate of cross-contamination. First, environmental sampling and bacteriological assessment were not performed systematically for every companion animal that presented to the ophthalmology service with an ocular infection. Instead, only bacterial isolates that were considered MDR prompted an environmental culture in the retrospective study, while only 23 patients were selected for corneal and environmental sampling during the prospective study. Second, environmental surveillance triggered by MDR cases was performed several days after the clinical patient was examined (i.e., once corneal culture results were available); therefore, routine cleaning of examination rooms may have yielded false-negative results in selected cases. Last, multiple swabs were lumped into “environment” and “equipment” groups in the prospective surveillance sessions due to cost constraints, limiting our understanding of specific sites of contamination for targeted disinfection. Of note, enrichment cultures were not performed in the present study as the culture media used (blood agar, MacConkey agar) were deemed sufficient to grow the majority of isolates considered pathogenic in canine eyes (e.g., *S. pseudintermedius, Streptococcus canis, Pseudomonas aeruginosa*) (9). Nonetheless, the use of enriched media could be considered in future studies to identify a greater variety of microbials involved in cross-contamination.

In summary, the present study reported a contamination rate of 32.9% in ophthalmic exam rooms at the authors' institution, describing the recovery of genetically indistinguishable isolates between the patient's cornea and surrounding environment in a subset of cases. Veterinary practitioners should be aware that contamination of the examination room (environment, equipment) occurs at a relatively high frequency (32.9%) following ophthalmic examinations in companion animals, potentially resulting in nosocomial infections due to cross-contamination between the patient and its surroundings. We hope this knowledge will enhance mitigation strategies (e.g., updating cleaning protocols) to reduce the risk of healthcare-associated infections in veterinary practices. Future studies could determine the effect of different cleaning protocols on bacteria recovery, identify specific locations within exam rooms that are most commonly contaminated, and consider sampling clinicians' hands following interactions with patients and manipulation of ophthalmic equipment.

## Data Availability Statement

The raw data supporting the conclusions of this article will be made available by the authors, without undue reservation.

## Ethics Statement

The animal study was reviewed and approved by the Ethics Committee of Lloyd Veterinary Medical Center. Written informed consent was obtained from the owners for the participation of their animals in this study.

## Author Contributions

DG, RA, and LS designed the study, performed the clinical experiments, and analyzed the data. OS, MA, and DK completed the laboratory experiments. All authors wrote the manuscript.

## Conflict of Interest

The authors declare that the research was conducted in the absence of any commercial or financial relationships that could be construed as a potential conflict of interest.
